# TAMGeS: a Three-Array Method for Genotyping of SNPs by a dual-colour approach

**DOI:** 10.1186/1471-2164-8-10

**Published:** 2007-01-09

**Authors:** Arianna Cozza, Francesco Morandin, Silvia Giulia Galfrè, Veronica Mariotti, Roberto Marangoni, Silvia Pellegrini

**Affiliations:** 1 Department of Experimental Pathology, Medical Biotechnology, Infectivology and Epidemiology, University of Pisa, Via Roma 55, 56126 Pisa, Italy; 2 Department of Mathematics, University of Parma, Parco Area delle Scienze 53/A, 43100 Parma, Italy; 3 Department of Informatics, University of Pisa, Largo Bruno Pontecorvo 3, 56127 Pisa, Italy

## Abstract

**Background:**

Many of the most effective high-throughput protocols for SNP genotyping employ microarrays. Genotypes are assessed by comparing the signal intensities that derive from the hybridization of different allele-specific probes labelled either by using four fluorescent dyes, one for each base, or by using only two dyes and investigating the polymorphic alleles two by two on separate arrays. The employment of only two dyes makes it possible to use a dual-laser scanner, which has the advantage of being present in every microarray laboratory. However, this protocol may present some drawbacks. To infer all the six possible genotypes it is necessary to compare signals from two arrays, but this comparison not always is successful. A number of systematic errors in the experimental protocol, in fact, may differently affect signal intensities on separate arrays. Here we present TAMGeS (Three-Array Method for Genotyping of SNPs), an exhaustive method for SNP genotyping through SBE (Single Base Extension) and dual-colour microarrays, which makes the comparison of signals on distinct arrays reliable by using a third array and a data handling method for signal normalization based on bilinear regression theory.

**Results:**

We tested the effectiveness of the proposed method by evaluating the results obtained from the direct comparison of the two arrays or by applying TAMGeS, both on experimental and synthetic data. With synthetic data, TAMGeS reduced the frequency of errors by an order of magnitude, when the incidence of systematic errors was not negligible. With the experimental data, produced by genotyping 25 SNPs in 437 subjects, TAMGeS reduced the percentage of missing genotypes from 54% (Two-Array Method) to 14.5%. Allelic and genotypic call rates were 99.3% and 99.5%, respectively. The normalization procedure takes into account also systematic errors, which can be generated by a time-delayed assay, thus making the protocol more flexible.

**Conclusion:**

TAMGeS represents an innovative method, which proved to be very effective in producing reliable SNP genotyping data by dual-colour microarrays. The requirement of a third array is well balanced by the strong enhancement in data quality and by the greater flexibility of the experimental protocol.

## Background

Genetic variations cause many phenotypic differences in the organism development and physiology, and contribute to individual disease susceptibility and drug response as well. The most widespread genetic variations are SNPs (Single Nucleotide Polymorphisms) that are frequently used in case-control studies to identify possible associations between genotypes and complex diseases [1,2].

At the present time, more than 10 million human SNPs are listed in the public databases, and this number is growing steadily. The amount of SNPs needed to obtain reliable results in association studies depends on linkage disequilibrium among them [3] and on their organization in haplotypes [4]. The ongoing identification of tagging-SNPs and haplotype blocks by the International HapMap Project [5] will make it possible to use smaller numbers of SNPs to capture all the genetic variation in a given chromosomal region [5,6]. Currently, however, many SNPs are needed to achieve this goal with statistically significant results. Since the DNA samples to test must also be numerous, it is essential to carry out such investigations in a high-throughput way.

In the last few years great efforts have been made in developing high-throughput methodologies for SNP genotyping in order to attain high sensitivity, low error rates and affordable costs [7-11]. Among these methods, several of the most effective relay on the use of microarrays [11-16]. Genotypes are assessed by comparing to each other the signal intensities [17] of hybridized allele-specific probes, which may be labelled either by SBE (Single Base Extension) [18-20], or by selective ligation (i.e., padlock probes) [14,21], or by allele-specific primer extension on microarray [22]. The fluorescent labelling of the polymorphic alleles may be carried out by: 1) conjugating each of the four bases to a different dye, with the mandatory use of a tetra-laser scanner for the acquisition of the microarray images [14,18]; 2) employing only two dyes [19,23], for example by hybridizing the labelled alleles two by two (e.g. A/C or G/T) onto distinct arrays [24] and using a dual-laser scanner to acquire the slide images.

Although powerful, the tetra-colour approach is very expensive, since four different fluorescent dyes are required and the tetra-laser scanner has very high acquisition and maintenance costs. On the contrary, the dual-colour approach is more easily affordable for all the microarray laboratories that already perform gene expression analysis, but it may present some drawbacks. Since each array is used to test directly only two bases (hence, we refer to them as partial arrays, *P*_*1 *_and *P*_*2*_), the signal intensities on the *P*_*1*_-array must be compared to those on the *P*_*2*_-array in order to infer all the six possible bi-allelic genotypes. However, genotype assignments through the direct comparison between arrays *P*_*1 *_and *P*_*2 *_are not completely reliable. A number of systematic errors, introduced at several steps in the experimental protocol (e.g., different labelling efficiency, differential binding rates of labelled products to different arrays, etc.), may differently affect the signal intensities on the two arrays, thus making their comparison unsuccessful.

To improve the dual-colour microarray approach for SNP genotyping, a normalization method, which produces coefficients able to make signal intensities from different arrays comparable, is needed. Such a method must take into account both systematic and random errors in the experimental procedure. Unfortunately, theoretical methods for an *a priori *normalization require a formal and quantitative description of each source of noise, which is not always feasible.

Here we propose TAMGeS (Three-Array Method for Genotyping of SNPs) as an alternative normalization approach based on bilinear regression theory, which requires a further experimental contribution: in addition to the two P-arrays, an extra array identical to the others (called *U *for Union) is hybridized with the products of a third labelling reaction in which each dye is conjugated with two bases. The signal intensities recorded from the *U*-array can be used to extract the normalization coefficients crucial to make the data recorded from the two P-arrays directly and reliably comparable.

We also describe software modules which we developed for data processing and analysis obtained by applying the proposed experimental protocol.

## Results

### Experimental approach

The DNA sequences containing the SNP loci were amplified by multiplex PCR (for primer sequences see Table 1); cyclic SBE reactions were carried out in the presence of fluorescently labelled ddNTPs by using primers with 3'- end complementary to the nucleotide exactly before the SNP and with a tag sequence at their 5'- end (for SBE primer sequences see Table 2). The SBE products were then hybridized onto the arrays to unique anti-tag probes complementary to the primer 5' tags. After slide image scanning, alleles and genotypes were assigned by software analysis.

### Two-Array Method

The simplest approach to execute SNP genotyping by microarray with only two dyes is the performance of two SBE reactions (*P*_*1 *_and *P*_*2*_), subsequently hybridized on distinct arrays [24]. We performed a *P*_*1 *_reaction with Cy5-ddATP and Cy3-ddGTP (and cold ddCTP and ddTTP) for the direct investigation of A/G polymorphisms, and a *P*_*2 *_reaction with Cy5-ddCTP and Cy3-ddUTP (and cold ddATP and ddGTP) for the direct investigation of C/T polymorphisms. The evaluation of the A/C, A/T, G/C and G/T genotypes was achieved through the comparison of the signals corresponding to the same SNP recorded from the two P-arrays.

The analysis of both P-arrays aimed at discriminating between true and false signals. For each spot, the background intensity values in the green (*b*_*g*_) and red (*b*_*r*_) channels and the distribution of green and red intensity values were extracted. It is generally accepted, in fact, that a signal may be identified as definitely true when its foreground value is several standard deviations above its background value. A direct consequence of this assumption is that signals in the range of the background value are classified as artefacts. It is not unlikely, however, to find a class of values statistically distant from the background, but showing values significantly lower when compared to top-score signals. As a consequence, the genotype assignment relative to ambiguous signals is a problem even on a single array. In the present case, another difficulty emerged, triggered by the need to compare the signals coming from two distinct arrays. Within any array, systematic comparison errors can be easily introduced by experimental random drifts; drifts can be caused by different global performances of the SBE probes due to different labelling efficiency or hybridization rate to the target on the array, as well as by a better or worse yield of each fluorescent dye.

In spite of an accurate optimization of the experimental protocol, also through a careful selection of primers for the multiplex PCR and for the SBE reaction, some inconsistencies in the procedure still remained, thus making genotype assignments not always reliable.

"Ambiguous signals", in fact, are very difficult to assign as true or false, since their relative error is maximum. As a consequence, instead of trying an *a priori *standardization of the data measured on the two P-arrays, which may prove to be unfeasible, we introduced a further data source, the *U*-array.

### TAMGeS (Three-Array Method for Genotyping of SNPs)

We hybridized the union array (*U*-array) with the products of a SBE reaction performed using all the labelled bases employed for the two P-arrays (Cy5-ddATP, Cy3-ddGTP, Cy5-ddCTP and Cy3-ddUTP). Therefore, for the *U*-array, the green signal was not associated to a single base, but to a pair of bases, the green-labelled in the *P*_*1*_-array and the green-labelled in the *P*_*2*_-array. The same was true for the red signal.

The *U*-array provided us with a supplemental source of experimental data, which can be deconvolved by using a bilinear regression approach (see additional file 1: TAMGeS theoretical basis). The signals deriving from the *U*-array enabled us to obtain an exact estimation of the ratio between the arrays *P*_*1 *_and *P*_*2*_, thus allowing for the direct comparison of their normalized signals and making the identification of A/T, A/C, G/C and G/T SNPs more efficient.

### Comparison between Two-Array Method and TAMGeS

In order to evaluate if TAMGeS increases the quality of SNP genotyping data obtained by dual-colour microarrays, we estimated the frequencies of genotyping errors derived from the direct comparison of the two P-arrays (Two-Array Method) or by using TAMGeS. We first exploited the performances of TAMGeS on a limited number of experimental data and then we expanded the number of samples by synthetic data.

#### Results from experimental data

**Figure 1:**
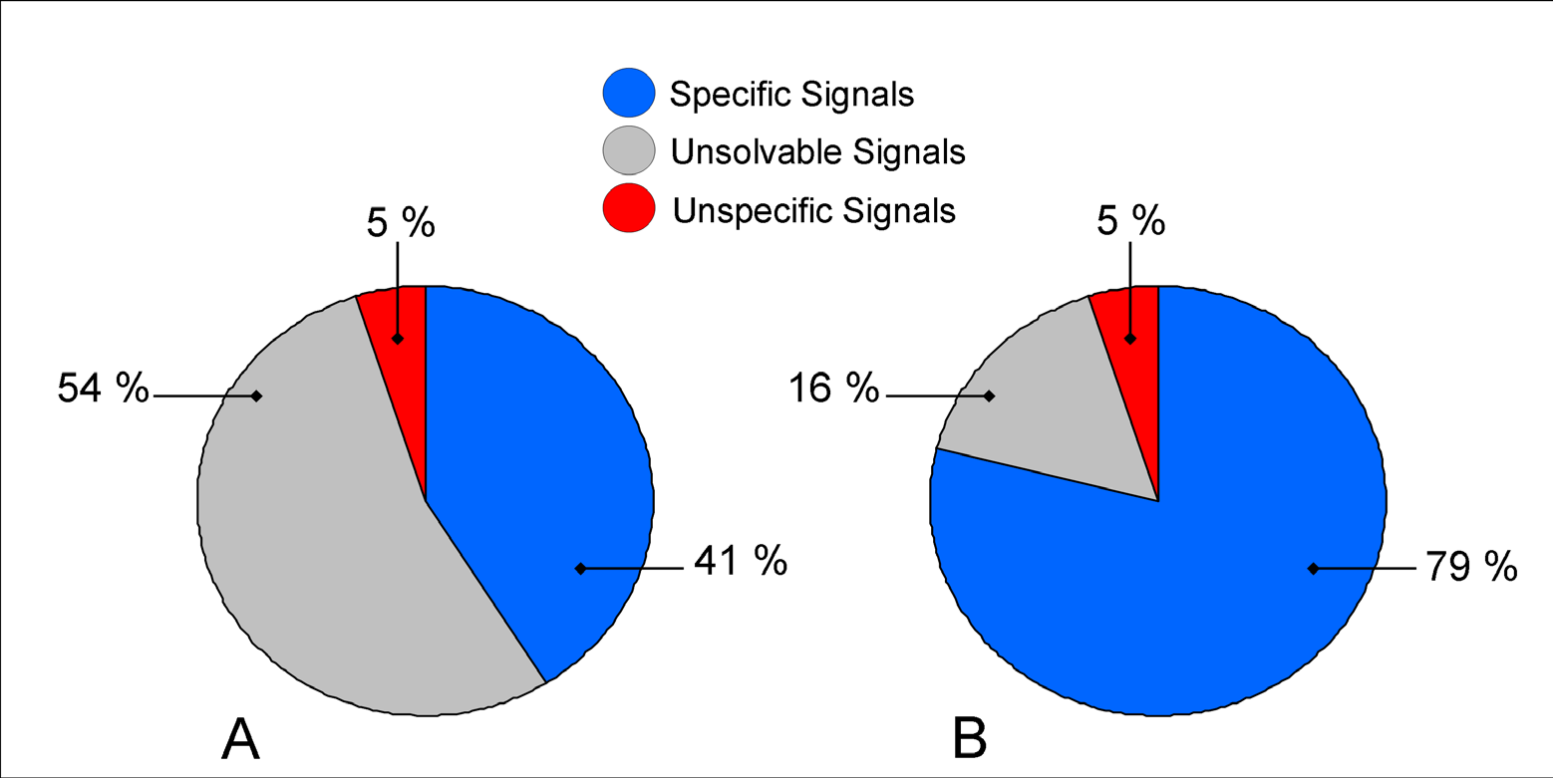
**Comparison between Two-Array Method and TAMGeS**. The comparison between the genotyping results of 87 samples (2,175 SNPs) by both Two-Array Method (A) and TAMGeS (B) is reported. With TAMGeS a striking reduction of the ambiguous signals (i.e. signals for which genotype assignment proves to be unfeasible, hence considered as missing signals) recovered as specific signals, can be observed. The percentage of unspecific signals (i.e. signals recorded in correspondence of alleles which result impossible according to the information given by the databases) is the same employing both the methods.

We compared the application of the Two-Array Method and TAMGeS by genotyping 25 SNPs (Table 3) in 87 samples for a total of 2,175 SNPs (Fig. 1).

With the Two-Array Method (Fig. 1A), the amount of missing data was very high: indeed, 54% of the signals came out as ambiguous (i.e., signals for which genotype assignment proved to be unfeasible). Moreover, 5% of the signals were recognized as unspecific (i.e., signals recorded for alleles to be considered as impossible according to the information given by the NCBI SNP database [25]). Thus, only 41% of the SNPs were assigned by this system. By analyzing the same 87 samples by TAMGeS, in contrast, ambiguous signals were only 16%, while the amount of unspecific signals remained 5% (Fig. 1B), so that 79% of the examined SNPs were assigned. The concordance rate between the 41% and 79% of SNPs, called by the two methods respectively, was absolute in the 77% of the cases and partial (one allele) in the 20% of the cases. The totally discordant genotypes accounted for the 3% of the cases.

The signals that remained ambiguous after the application of TAMGeS included both signals with foreground intensity values lower than the background intensity value and signals discarded in the data processing because the sum of their normalized intensities on the two P-arrays was not equal to their intensity on the *U*-array (see additional file 1: TAMGeS theoretical basis, Extracting information from the *U*-array).

These results suggest that the application of TAMGeS not only allows for the reduction of the uninterpretable signals, but also increases the confidence in the assigned genotypes. Applying both the two- and the three-array methods, we noticed that the unspecific signals (5%) were related to the same few SNPs, and always restricted to one channel, probably accounting for non-random phenomenon of cross-reaction to unspecific templates.

Moreover, the normalization procedure takes into account systematic errors which can be generated by a time-delayed assay and is able to filter them. If any array (*P*_*1*_, *P*_*2*_, *U*) fails in producing analyzable signals, it can be recovered by simply hybridizing the SBE products on another slide, or by repeating only the failing SBE reaction.

#### Results from synthetic data

To strengthen the results obtained with experimental data, we generated synthetic data by simulating a typical SNP genotyping experiment according to the procedure applied to obtain real data.

We generated a total of 2,880 random experiments, each one with 750 samples over a set of 100 SNPs. We set the frequency of both alleles of each SNP equal to 0.5, and we computed the frequency of errors only among the samples/SNPs with two signals. Each simulated experiment exhibited some unique random (e.g., each SNP having a typical intensity, and a distinctive amount of artefact signals which can give rise to false heterozygous genotypes) and some deterministic parameters. Most of these parameters were generated by looking at our preliminary experimental data drawn from the Two-Array Method, but we analyzed in detail the only two which we considered as potentially critical for the comparison: the global frequency of artefacts and the multiplicative variability between the two arrays. The 2,880 experiments were subdivided into 6 levels of frequency (from 5% to 30%) and 16 levels of variability (from 0 to ×4). We performed 30 simulations for each experiment.

**Figure 2:**
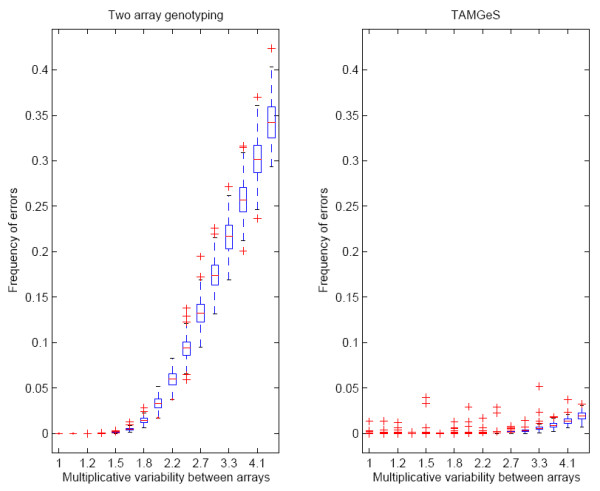
**Box plots of genotyping error frequency computed on synthetic data**. Both graphs report on the abscissa the multiplicative variability between the two arrays, and on the ordinate the frequency of genotyping errors. The graph on the left shows the results obtained using the direct Two-Array Method; the one on the right, the results obtained using TAMGeS. The greater robustness of TAMGeS is clearly evident.

**Figure 3:**
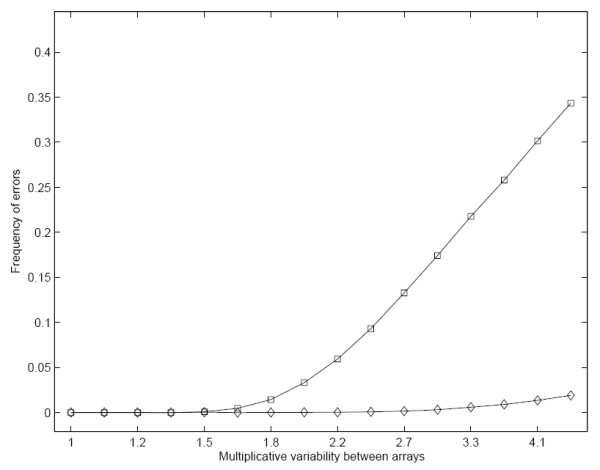
**Average frequency of errors *vs*. multiplicative variability computed on synthetic data**. This plot shows on the X axis the multiplicative variability between the arrays, and on the Y axis the average value of the frequency of genotyping errors. Symbols indicate the methods: squares refer to the direct Two-Array Method; diamonds refer to TAMGeS. For multiplicative variability greater than 1.8, the genotyping performance of TAMGeS results significantly better than that of direct Two-Array Method.

The results obtained on synthetic data are shown in Figures 2 and 3; in both the figures, the 6 levels of artefact frequency were collapsed together, since no qualitative difference emerged from different levels. Figure 2 shows the box-plots of the 180 data corresponding to the different levels of multiplicative variability. Figure 3 shows the averages of the same data in a single plot. It is evident that if the variability between arrays is small, there is no need at all to normalize them through the third array: outliers in the right part of Figure 2 (referring to TAMGeS) show that sometimes (rarely) the regression fails to determine the right coefficients and the frequency of error increases. On the other hand, if the variability is high (> 1.8), the Two-Array Method becomes very faulty, while TAMGeS reduces the frequency of errors by one order of magnitude (Fig. 3).

### Evaluation and validation of TAMGeS

To estimate the global efficiency of the proposed genotyping method, we assessed the amount of missing data by experimentally analyzing 350 additional samples. For all the 25 SNPs, ambiguous signals accounted for 14.5% on average, ranging from 5.3% to 29.9% for different SNPs (see additional file 2: Graphs, Graph 1 – Unsolvable signals). Among all the analyzable signals, we verified that non-assigned alleles were only 0.7% on average, ranging from 0% to 2.5% for different SNPs (see additional file 2: Graphs, Graph 2 – Non-assigned alleles). Non-assigned genotypes were 0.5% on average, ranging from 0% to 2.5% for different SNPs (see additional file 2: Graphs, Graph 3 – Non-assigned genotypes).

To estimate the reliability of our method, we sequenced four DNA samples for each SNP by an ABI Prism 310 Genetic Analyzer (Applied-Biosystems). We obtained concordant results with the exception of four SNPs for which one allele was missed in one sample. Interestingly, our method allows for a clear discrimination between heterozygous deletion genotypes (A/-) from homozygous genotypes (A/A), whereas the sequencing gives the same pattern in both the cases.

All the analyzed SNPs were tested by hwsim.exe [26] and resulted to be in Hardy-Weinberg equilibrium.

## Discussion

TAMGeS genotypes all the possible SNPs by performing three SBE reactions (*P*_*1*_, *P*_*2 *_and *U*) with different combinations of labelled ddNTPs, hybridized on three identical arrays. Other dual-colour approaches request either a distinct array for each of the possible base changes or the use of one labelled allele-specific probe for each SNP [21,23,27].

Most of the existing methods, based both on tetra- and dual-colour approaches, do not succeed in genotyping all the SNPs with equal efficiency; SNPs with a too high data loss are simply discarded from the analysis. If many SNPs are analysed, as in large scale studies or wide genome scans, such a loss of data does not usually compromise the overall informative power of the study. On the contrary, in the context of association studies of candidate genes and SNPs, retrieving maximum information is essential. Our approach guarantees acquisition of reliable data also in those cases where SNP genotyping proves to be difficult. Indeed, with the three-array approach the overall amount of unsolvable signals was 14.5%, thus significantly smaller as compared to 54% obtained by the previously employed two-array approach, and lower than the percentage of SNPs usually discarded by other methods [14,18,28]. In addition, TAMGeS resulted more accurate in calling genotypes than the Two-Array method. The concordance rate between the two approaches, in fact, was not absolute (77% of the cases with both the alleles in common and 20% of the cases with only one allele), while the concordance rate between TAMGeS and the sequencing method was almost absolute. Thus, the increased cost due to the use of the third array is well balanced by this strong enhancement in data quality.

Spots on distinct arrays may show very different signal intensities because each array may be considered as a microenvironment separate from the others. We observed that slide printing quality, for example, may determine as much as 20% variability in signal intensities between twin spots and as much as 30% variability between the same spots in replicated samples. Solving the variability between the arrays *P*_*1 *_and *P*_*2 *_might account for the observed decrease of unsolvable signals. The introduction of the *U*-array and the application of a bilinear weighted-least-square regression model (see additional file 1: TAMGeS theoretical basis) allow for the calculation of normalization factors for the arrays *P*_*1 *_and *P*_*2 *_and, consequently, for their direct and unambiguous comparison.

The percentage of residual unsolvable data resulted to be different for each SNP, ranging from about 5% to 30%. Missing data proved to be mostly dependent on the unequal efficiency of the SBE primers, either in the SBE reaction or during the hybridization, which produced signals below the detection threshold. In the SBE reaction, the efficiency in the incorporation of labelled ddNTPs might be affected by secondary structures in the template, as we verified for several SNPs by Mfold 3.1 software [29]. As far as the hybridization is concerned, too low signals might depend on the investigating anti-tags; anti-tags can be poorly spotted or too close to the hybridization positive control, which usually has a strong signal spreading in the surrounding background. A more careful selection of efficient primers and utilisation of only good quality slides would likely decrease further the amount of missing data. We got an overall allelic and genotypic call rate respectively of 99.3% and 99.5% on average.

We adopted a multiple statistical approach (*standard*, *new *and *average*) for analyzing data as in a case-control association study (see Methods, Data analysis, *Fourth Module: statistical analysis*). This approach conferred consistency to genotyping and association data since the three distinct p-values deriving from each approach were concordant. Moreover, the *new *and *average *analyses, taking into account the probability with which genotypes and alleles are assigned, weighted the confidence of the data.

It is noteworthy that all the obtained genotyping data were certain, as established by sequencing validation. Moreover, the fact that all the SNPs were in Hardy-Weinberg equilibrium confirmed the coherence of the proposed system [30].

The application of TAMGeS confers an important experimental advantage: the ruling-out of the necessity to perform all the steps of the protocol, from the DNA target amplification to the hybridization on microarray, at the same time for both the P-arrays. For a given sample, indeed, TAMGeS is cost-effective in reclaiming partial data due, for example, to the failure of at least one out of the three arrays: relying on the normalization peculiarities of the *U*-array, only the failed array is recovered as long as the SBE reaction is performed on the same PCR product. With the two-array approach, if only one array fails, it is necessary to re-hybridize both, since the variability would be too high to be neglected. This means that TAMGeS makes the two-colour microarray protocol for SNP genotyping not only more reliable, but also more flexible.

## Conclusion

TAMGeS represents a useful, flexible and high-throughput tool for SNP genotyping by dual-colour microarrays, which enables laboratories equipped with dual-laser scanners, usually employed for gene expression studies on microarray, to perform also SNP genotyping without any additional requirement or costs.

The main advantages of TAMGeS, compared to other existing dual-colour approaches, are:

1) cost-effectiveness due to:

a. the utilization of non labelled allele-specific primers and probes,

b. the use of a reduced numbers of arrays (three instead of six),

c. the possibility of repeating just one array, instead of all those relative to a sample, in the event that a single array has failed;

2) higher specificity due to the multiple statistical approach employed for the correlation analysis, which allows for the estimation of the confidence of the obtained data.

This is the reason why TAMGeS is especially suited for association studies based on candidate genes and SNPs, where the number of SNPs to be genotyped is limited and achieving the highest possible knowledge from all the selected SNPs is an essential goal.

## Methods

### DNA samples

DNA samples were collected after obtaining a written, informed consent from each of the subjects. The study was conducted in accordance with the provisions of the Helsinki Declaration and according to a protocol approved by the Ethics Committee of the University of Florence.

### SNP selection

We selected 25 SNPs in 5 genes (Table 3) from the NCBI dbSNP [25], according to this order of criteria: 1) SNPs determining a not synonymous amino-acidic change; 2) SNPs located in promoter regions, putative splice sites or untranslated regions; 3) validated SNPs.

### Multiplex PCR and SBE reaction

For each sample, the sequences containing the SNPs were amplified by QIAGEN^® ^Multiplex PCR Kit (QIAGEN) from genomic DNA (40 ng in each reaction) in two 25-μl multiplex PCR mixes. Amplification conditions were as follows: hot-start at 95°C for 15 min; 30 cycles of 30 s at 94°C, 1 min and 30 s at 63°C, 1 min at 72°C; final elongation at 72°C for 10 min. Products of the two PCRs were pooled and incubated for 1 h at 37°C with 16 U of exonuclease I (USB-Corporation) and 1.6 U of shrimp alkaline phosphatase (USB-Corporation), then inactivated for 5 min at 65°C. Total yield of each amplified product was assessed after purification by MinElute PCR Purification Kit (QIAGEN). 1.5 μl of purified products were used as templates in a 30-μl SBE reaction, performed with all the SBE primers, 3.5 U of Thermo Sequenase™ DNA Polymerase (GE-Healthcare), 1X TS reaction buffer, the proper combination of cold (GE-Healthcare) and/or labelled ddNTPs (Perkin-Elmer) (0.125 μM each) (see Results). Cycling conditions were: hot-start at 92°C for 3 min and 30 s; 40 cycles of 45 s at 92°C and 30 s at 63°C.

PCR (Table 1) and SBE primers (Table 2) were designed by Genamics Expression 1.1 [31] and purchased from MWG-Biotech AG. We selected PCR primers aimed at minimizing formations of cross- and self-dimers; their specificity was tested by BLAST [32]. Primer amounts were scaled up and down to find out the concentrations which allowed for the amplification of all the expected fragments with equal efficiency, taking into account the number of SNPs in any sequence. Concerning the SBE primers, we used the one, between forward and reverse, which minimized hairpin and self-dimer formations. For those SNPs a few bases away from each other, we chose primers on opposite strands in order to avoid reciprocal interferences in the extension step. We tested matches between tags and SBE primers and we opted for the combinations avoiding self- and cross- secondary structures, as well as the annealing to unspecific templates.

### Microarray hybridization and data acquisition

**Figure 4:**
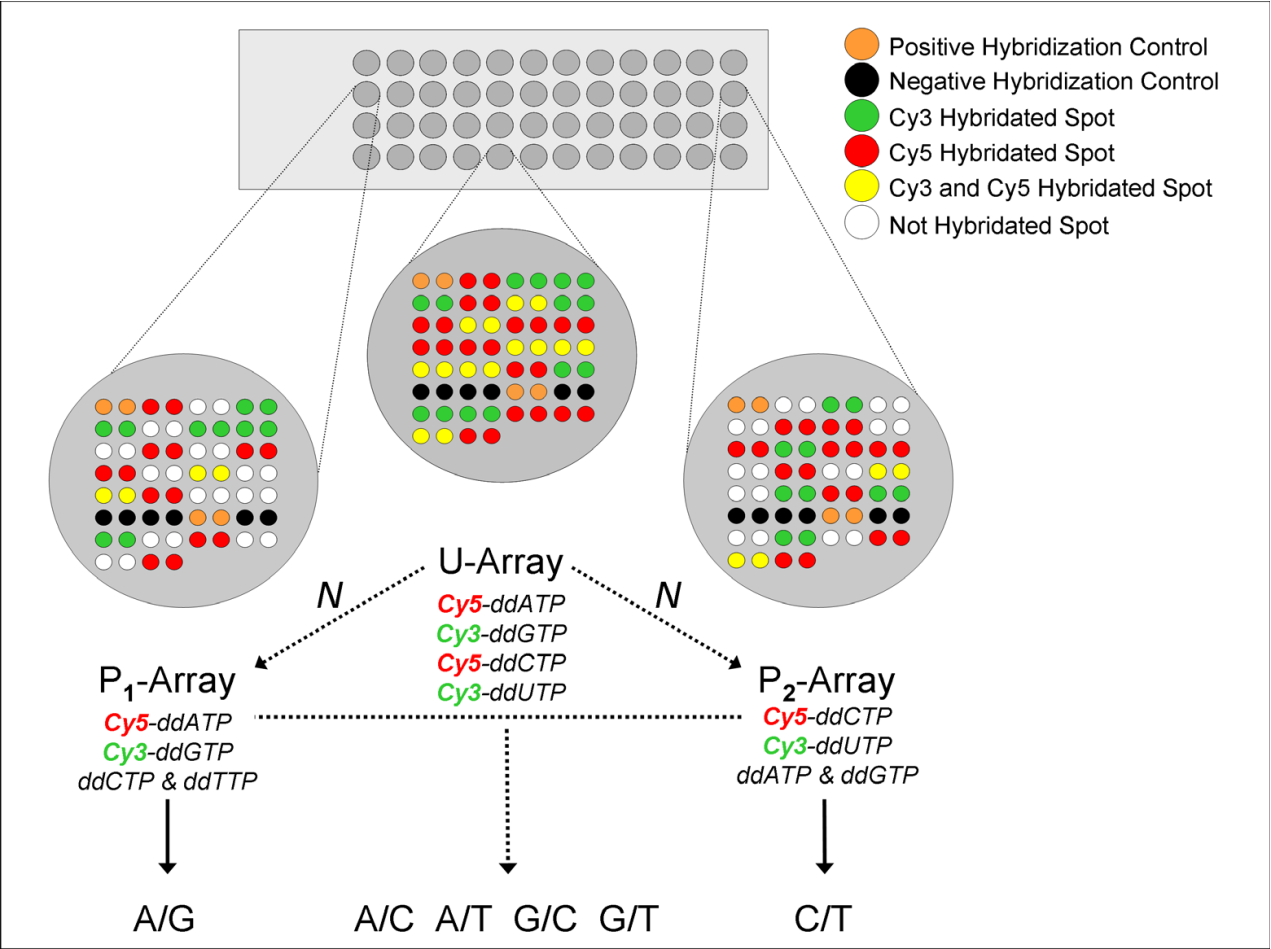
**SNP genotyping by TAMGeS**. On the upper left an image of our microarrays (tagged "array of arrays" format) on which 48 separate reaction chambers are created. On three distinct arrays (*P*_*1*_, *P*_*2 *_and *U*), we hybridize the SBE reactions performed on each DNA sample with three different combinations of Cy3 or Cy5 labelled ddNTPs. The *U*-array is exploited to normalize (***N***) signals from arrays *P*_*1 *_and *P*_*2*_, in order to compare them directly (dashed lines) and to genotype all the possible polymorphic alleles.

Microarray slides, purchased from Leiden Genome Technology Center (LGTC, University of Leiden), were printed in an "array-of-arrays" configuration, including 48 identical arrays (Fig. 4) which allow the contemporary genotyping of 16 samples. Twenty-nine anti-tag probes (20-mer long), selected from the universal GenFlex^® ^Tag Array set (Affymetrix P/N 610026), were spotted in duplicate on each array and were used to detect 25 SNPs, a couple of identical positive hybridization controls and three negative hybridization controls (Fig. 4).

Before the hybridization, the slides were treated with 120-μl of pre-hybridization solution (warmed at 65°C), containing 48 μg herring sperm DNA (Gibco-BRL), 48 μg yeast tRNA (Gibco-BRL), 48 μg polyA-RNA (Sigma), 5X Denhardt's solution, 0.4% sodium dodecyl sulphate (SDS), 3.5X SSC (20X SSC is 3.0 M Sodium Chloride, 0.3 M Sodium Citrate, pH 7.0). The slides were covered with a cover-slip (sealing on a hot-plate at 80°C for 2 min), then kept at 65°C for 30 min in a dark and wet chamber. Cover-slip was removed in 2X SSC (2 min) and slides were next washed in 2X SSC, 70% ethanol twice, 90% ethanol, 100% ethanol (5 min each step) and dried by spinning (3 min at 250 × *g*).

For the hybridization, the slides were located in a custom-made aluminium rack and a silicon rubber grid was placed over each slide to create 48 reaction chambers in correspondence of the arrays. A 25-μl mix, containing 10 μl of SBE reaction, 6X SSC and 0.27 nM of two positive hybridization control probes (labelled in 5' with Cy3 or Cy5) (MWG-BiotechAG), was injected into each chamber. The hybridization was carried out at 42°C for 5–16 h. After disassembling the rack in 4X SSC, the slides were washed (5 min each step) twice in 2X SSC, 0.1% SDS (warmed at 40°C) and twice in 0.2X SSC, briefly rinsed in deionized water and spin-dried in the dark.

Slide scanning was performed by GenePix 4000B dual-laser scanner (Axon-Instruments). Signal intensity values were extracted by GenePix Pro 4.0 (Axon-Instruments).

### Data analysis

Data processing and statistical analyses were carried out by software, written in four independent modules, which we developed accordingly with the proposed protocol. We split the samples into three groups (#1, #2 and reference group) in order to perform correlation analysis, as in a case-control association study.

#### First module: colour balancing and signal counting

As a first step, the software regards a spot (*i*) as analyzable, i.e. correctly hybridized, if at least 70% of its pixels (foreground) have an intensity (*I*) higher than one standard deviation above the background intensity for at least one channel (red, R; green, G). Then, the intensity value of each spot is corrected by the red/green intensity ratio, a colour scaling factor which accounts for the scanner sensitivity and the labelling efficiency. For a whole slide the red/green ratio is calculated as I¯R/I¯G
 MathType@MTEF@5@5@+=feaafiart1ev1aaatCvAUfKttLearuWrP9MDH5MBPbIqV92AaeXatLxBI9gBaebbnrfifHhDYfgasaacH8akY=wiFfYdH8Gipec8Eeeu0xXdbba9frFj0=OqFfea0dXdd9vqai=hGuQ8kuc9pgc9s8qqaq=dirpe0xb9q8qiLsFr0=vr0=vr0dc8meaabaqaciaacaGaaeqabaqabeGadaaakeaadaGcaaqaaiqbdMeajzaaraWaaSbaaSqaaiabdkfasbqabaGccqGGVaWlcuWGjbqsgaqeamaaBaaaleaacqWGhbWraeqaaaqabaaaaa@32AE@, where I¯
 MathType@MTEF@5@5@+=feaafiart1ev1aaatCvAUfKttLearuWrP9MDH5MBPbIqV92AaeXatLxBI9gBaebbnrfifHhDYfgasaacH8akY=wiFfYdH8Gipec8Eeeu0xXdbba9frFj0=OqFfea0dXdd9vqai=hGuQ8kuc9pgc9s8qqaq=dirpe0xb9q8qiLsFr0=vr0=vr0dc8meaabaqaciaacaGaaeqabaqabeGadaaakeaacuWGjbqsgaqeaaaa@2DDF@_*R *_is (1/n⋅∑iG=0nIRiG
 MathType@MTEF@5@5@+=feaafiart1ev1aaatCvAUfKttLearuWrP9MDH5MBPbIqV92AaeXatLxBI9gBaebbnrfifHhDYfgasaacH8akY=wiFfYdH8Gipec8Eeeu0xXdbba9frFj0=OqFfea0dXdd9vqai=hGuQ8kuc9pgc9s8qqaq=dirpe0xb9q8qiLsFr0=vr0=vr0dc8meaabaqaciaacaGaaeqabaqabeGadaaakeaacqaIXaqmcqGGVaWlcqWGUbGBcqGHflY1daaeWbqaaiabdMeajnaaBaaaleaacqWGsbGudaWgaaadbaGaemyAaK2aaSbaaeaacqWGhbWraeqaaaqabaaaleqaaaqaaiabdMgaPnaaBaaameaacqWGhbWraeqaaSGaeyypa0JaeGimaadabaGaemOBa4ganiabggHiLdaaaa@3FAA@), i.e. the average intensity of the red channel calculated on all the spots which result analyzable in the green channel (*i*_*G*_), and I¯
 MathType@MTEF@5@5@+=feaafiart1ev1aaatCvAUfKttLearuWrP9MDH5MBPbIqV92AaeXatLxBI9gBaebbnrfifHhDYfgasaacH8akY=wiFfYdH8Gipec8Eeeu0xXdbba9frFj0=OqFfea0dXdd9vqai=hGuQ8kuc9pgc9s8qqaq=dirpe0xb9q8qiLsFr0=vr0=vr0dc8meaabaqaciaacaGaaeqabaqabeGadaaakeaacuWGjbqsgaqeaaaa@2DDF@_*G *_is (1/n⋅∑iR=0nIGiR
 MathType@MTEF@5@5@+=feaafiart1ev1aaatCvAUfKttLearuWrP9MDH5MBPbIqV92AaeXatLxBI9gBaebbnrfifHhDYfgasaacH8akY=wiFfYdH8Gipec8Eeeu0xXdbba9frFj0=OqFfea0dXdd9vqai=hGuQ8kuc9pgc9s8qqaq=dirpe0xb9q8qiLsFr0=vr0=vr0dc8meaabaqaciaacaGaaeqabaqabeGadaaakeaacqaIXaqmcqGGVaWlcqWGUbGBcqGHflY1daaeWbqaaiabdMeajnaaBaaaleaacqWGhbWrdaWgaaadbaGaemyAaK2aaSbaaeaacqWGsbGuaeqaaaqabaaaleqaaaqaaiabdMgaPnaaBaaameaacqWGsbGuaeqaaSGaeyypa0JaeGimaadabaGaemOBa4ganiabggHiLdaaaa@3FC0@), i.e. the average intensity of the green channel calculated on all the spots which result analyzable in the red channel (*i*_*R*_). We considered the intensity of the other channel respect to the one for which a spot was analyzable, since this intensity could be either a real signal (for heterozygous SNPs) or background noise; therefore, the signal behaviour in both these situations (specific and unspecific signals) is taken into account.

For each array, the software counts in each channel how many analyzable spots have an average signal intensity value of at least 100. A given number of spots (half number of the expected hybridized spots) have to be counted to consider an array as successfully hybridized.

#### Second module: signal normalization

The second module of the software estimates the scaling factors for the signal intensities of the arrays *P*_*1 *_and *P*_*2*_; normalizing the arrays by these factors yields values comparable between them. The factors are calculated by bilinear weighted-least-squares regression model (see additional file 1: TAMGeS theoretical basis, Applying bilinear regression), which considers the signal intensities of the *U*-array as the sum of the signal intensities of the arrays *P*_*1 *_and *P*_*2*_. After correction, for each SNP the software verifies that the sum of the signal intensities from arrays *P*_*1 *_and *P*_*2 *_corresponds to the signal intensities from *U*-array; otherwise the SNP is considered lost.

#### Third module: genotype and allele assignment

**Figure 5:**
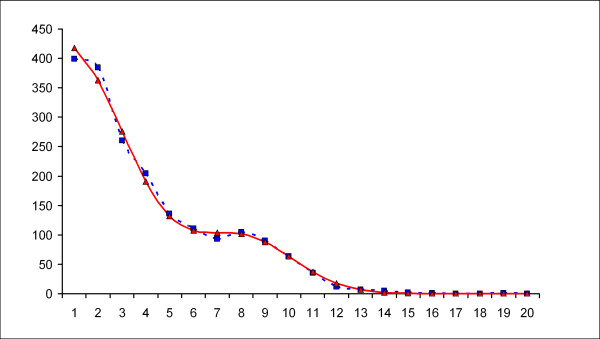
**Representation of Z-distribution**. The empirical distribution of the absolute value of the log-ratio of logarithms of intensities (Z) of all the signals recorded from the analyzed arrays is represented. On the x-axis the value of the variable Z is plotted; on the y-axis the value of the density function of the variable Z probability is reported. The Z-distribution appears as a bimodal density with two peaks, and it can be decomposed into two normal distributions: one with positive mean (see the peak on the right), representing the distribution of those SNPs with very different intensity values for the two alleles (homozygous genotypes), and the other with mean zero (see the peak on the left), representing the distribution of those SNPs with similar intensity values for both the alleles (heterozygous genotypes). The bold line (with filled triangles) represents the best fitting to a mixture of normal distributions obtained through an E-M algorithm, while the dashed line (with open squares) represents the real data.

For each SNP, the genotype can be assigned with a certain probability by comparing the intensity values (*I*_*A*_, *I*_*a*_) corresponding to the two possible alleles (*A*, *a*). We established the probability of each possible genotype following a scalar approach. The absolute value of the log-ratio between the logarithmic intensities, defined as Z = |ln (ln *I*_*A*_/ln *I*_*a*_)|, gives an index for the strength of the signal difference between the two bases and allows for the discrimination between a true heterozygote (lower values of Z) and an artefact lower signal on the other base (higher values of Z). The distribution of Z was plotted on the whole data set (Fig. 5) and appeared as a bimodal density obtained as a mixture of two distributions: one, normal with positive mean representing the distribution of those SNPs with distant values of *I*_*A *_and *I*_*a *_(homozygous genotypes, *AA *and *aa*), and the other, the positive half of a normal with mean zero representing the distribution of those SNPs with similar values of *I*_*A *_and *I*_*a *_(heterozygous genotype, *Aa*).

In order to compute the *a posteriori *probabilities with which a given SNP belongs to each of the two distributions (*AA *+ *aa *and *Aa*), a Bayesian test is applied explicitly decomposing Z as a mixture of them. The parameters of *AA *+ *aa *and *Aa *(*a priori *probabilities, mean and standard deviation) are calculated through a standard EM (Expectation-Maximization) algorithm, which turns out to converge in ~120 iteractions; SNP *a posteriori *probability is then computed.

Dropping the absolute value from |ln (ln *I*_*A*_/ln *I*_*a*_)|, a signed value of Z is obtained, which can be compared with the mixture of three normal distributions (*AA*, *Aa *and *aa*) by symmetrizing the original Z distribution. For each SNP, a probability for each of the three possible genotypes (*P(AA)*, *P(Aa)*, *P(aa)*, which sum up to 1) is therefore given. Two thresholds are set for univocally assigning the genotype (heterozygous if *P(Aa)*>0.5; homozygous if *P(AA)*>0.66 or *P(aa)*>0.66). There are two intervals of Z-values for which no genotype is assigned: they are symmetric with respect to zero and they correspond to the uncertain cases in which *P(Aa)*<0.5, *P(AA)*<0.66 and *P(aa)*<0.66. The software assigns at least one out of the two alleles if the sum of the probabilities of the genotypes containing that allele is at least 0.9 (allele *A*, if P(*AA*) + P(*Aa*)>0.9; allele *a*, if P(*aa*) + P(*Aa*)>0.9).

#### Fourth module: statistical analysis

To assess the correlation between the genotypes of every SNP and each group of subjects analysed (i.e., #1, #2, reference group), the software executes three different tests, named *standard*, *new *and *average*.

In the *standard *method, classical Fisher-Irwin tests are done on the allelic counts (group #1 vs. reference; group #2 vs. reference). As a drawback the information on how sure a genotype is gets lost: after applying the threshold, a 70% *AA *is the same as a 99% *AA*.

In the *new *and *average *methods, the probability of each genotype is taken into account. With the *average *method, a classical Fisher-Irwin test is applied on a *modified *allelic count, yielded by summing the *expected *number of alleles for each SNP in each group. Allele *A *average count is computed as ∑_*i*_[2*P*_*i*_(*AA*) + *P*_*i*_(*Aa*)], where *i *is the sample and the sum is over all the samples in the group. A sample *i *with probabilities of *AA*, *Aa *and *aa *respectively 0.70, 0.25 and 0.05, counts for 2·0.70 + 0.25 = 1.65 *A *alleles and 0.35 *a *alleles. In the *new *method, a string with the genotypes of all the samples is randomly generated, according to given probabilities and assuming independence between different samples. This is done 20,000 times: each time the software computes the p-value of the Fisher-Irwin test computed on the simulated genotypes; the median of these p-values is returned.

The three p-values (*standard*, *new *and *average*), corrected for multiple comparisons, by applying either the exact correction for independent tests 1 - (1 - *p*)^*n*^, or the Bonferroni correction *np *(here *n *is the number of the tests), turned out to be concordant.

## Authors' contributions

AC contributed to design the study, performed the experimental validation of the method and the data analysis, and drafted the manuscript. FM participated in the design of the study, performed the statistical analysis and contributed to draft the manuscript. SGG conceived and developed the theoretical basis of the proposed method and wrote the software modules. VM participated in organizing and carrying out the wet part of the work. RM supervised the theoretical part of the work and contributed to the writing of the manuscript. SP conceived and supervised the experimental validation of the method, contributed to the interpretation of the results and to the writing of the manuscript. All authors read and approved the final version of the manuscript.

## Supplementary Material

Additional File 1 TAMGeS theoretical basis. It is a PDF file and includes two sections, respectively entitled "Extracting information from the *U*-array" and "Applying bilinear regression", describing the mathematical foundations of the presented data handling method. Click here for file

Additional File 2 It is a PDF file, including three graphs respectively on amount of unsolvable signals, allelic and genotypic call rates: Graph 1 – Unsolvable signals. Graph 2 – Non-assigned alleles. Graph 3 – Non-assigned genotypes. Click here for file

## Figures and Tables

**Table 1:** Multiplex PCR primer pairs

***SNPs***	**SEQUENCE (*FORWARD *and *REVERSE*)**	**μM**	**PRODUCT LENGTH**
*rs3738701*	5'- ACCTGAGAGGGCAAGTCAGAACCAACT -3' 5'- CGAGGTCCTGTGTTCGGCAACTTTA -3'	0.16 0.16	197
*rs11466112*	5'- CAAAGCACTGGAACTCATATTGTACCACG -3' 5'- AATAATTTACAGGTTGAGGTAGGGAGGGG -3'	0.32 0.32	221
*X60202*	5'- TAATGAGACACCCACCGCTGCTGTG -3' 5'- TCTACCGGAGGGGAGGAAAGAAGGAGAC -3'	1.60 1.60	262
*rs741072* *rs734194*	5'- GAAGCATCGGAGGGAATTGAGGTCTG -3' 5'- GCCAAGCTCAGGAAAAAACAGTCCTTTG -3'	0.28 0.28	301
*rs2289656* *rs2289658*	5'- TGCCTAACAAATGAGATGGATGTCTTTCCT -3' 5'- GAACCCTCCACTCCTGAACCCTGAA -3'	0.24 0.40	325
*rs6336* *rs6339* *rs1799770*	5'- TGCCTCTACTGTTCTCTCAATCCTCCACTT -3' 5'- CCTGCTCATGCCAAAATCACCAATCTT -3'	0.24 0.24	352
*rs6265* *rs3750934* *rs8192466*	5'- GGGGAAACACTGCATGTCTCTGGTTTATAT -3' 5'- GACTACTGAGCATCACCCTGGACGTGTAC -3'	0.24 0.24	530

*rs2275857*	5'- AACCACCCTCCCTTCCTTTCTCTAGGTCTA -3' 5'- AACCCTTCTTACCTTTCATGCCAAACTTG -3'	0.16 0.16	136
*rs1047856*	5'- GCTTCAGTGGTTCTATAACGGGGCAATAT -3' 5'- CTCATCCTTCCCATACTCATTCTTGGCTATT -3'	0.32 0.32	171
*rs3753213*	5'- CACCGCCTAGTCCCTTGGTTCTGAC -3' 5'- GTGGCTGCACTAACCCATCCGTCTC -3'	1.44 1.44	291
*rs1800878*	5'- GCATGTGCATGTGTATTGTGAGGGAGTAAG -3' 5'- CTCAAAGCCCTGAGCTTCCTGACTTCTC -3'	0.32 0.32	328
*rs2072446* *rs2072445*	5'- GCCAGAGTCACCCAGCAAGTCAGTG -3' 5'- CCGTGCTGGCTATGAGGTCTTGTTCT -3'	0.16 0.16	347
*rs6330* *rs11466110* *rs11466111*	5'- TGCATAGCGTAATGTCCATGTTGTTCTACA -3' 5'- GATGATGACCGCTTGCTCCTGTGAGTC -3'	0.32 0.32	382
*rs1048218* *rs1048220* *rs1048221*	5'- AAGAGGCTTGACATCATTGGCTGACACT -3' 5'- CATGGGATTGCACTTGGTCTCGTAGA -3'	0.32 0.32	410

Primer pairs are subdivided according to the PCR multiplex mix (each performed with 7 primer pairs) in which they were used. For each pair, there are indicated the SNPs comprised in the corresponding amplified fragment, the primer sequence of the primers (*forward *and *reverse*), the concentration employed in the reaction and the length (in *bp*) of the corresponding amplified fragment.

**Table 2:** SBE primers

***SNP***	**SBE PRIMER SEQUENCE**
*control*	*5'- **CATGAGCTAGAAGTCAGGAC**ACCATGGTGCACCTGACTCCTG -3'
*rs6330*	5'- **CCACGCATCCAAGATTAGGA**GAATGCTGAAGTTTAGTCCAGTGG -3'
*rs3738701*	5'- **CGAGTGACTAGATACGCTAT**GGCTAGGGGAGCTGCATTT -3'
*rs11466112*	5'- **CATAGATGGAAATCGGCGCA**TGGCAAGCAGGCTGCCTGG -3'
*rs11466110*	5'- **AGAGACGACCTAAGCCAGTC**ACGGGGTGAACGGAGTCGC -3'
*rs11466111*	5'- **CGAGTGACTAGATACGCTAT**GGGGCAGACCCGCAACATTACT -3'
*rs6336*	5'- **TAACCGCTCGTCATGTGTCT**GCTTGGCATCGGGTCCAT -3'
*rs6339*	5'- **ACGGATTCAGCAGTCCTCAT**GGAGCCACATCCTCCCCA -3'
*rs1799770*	5'- **AGATGTAGAACACCCGTCAC**CTATCCCCTCTCCTTTTCTTGTTC -3'
*rs3753213*	5'- **AGATGCGTGAGCACTACTTG**GAGCCTCTAGGAGGTCGTCCTT -3'
*rs1800878*	5'- **CAGTCGGTAGAACTATGTA**CCCTCCCTGACCTTCTGGTCT -3'
*rs6265*	5'- **TGCCAGCTAAGACACAGTA**CCCTCATCCAACAGCTCTTCTATCA -3'
*X60202*	5'- **GCAAACGGGTTGTAATTCTG**GGGTCCACACAAACCTCACG -3'
*rs1048218*	5'- **AACGATCCGGTACATTTAGG**TCTTCATTGGGCCGAACTTT -3'
*rs1048220*	5'- **ATCAGGTCTGGAAGATTACG**TGCTGCAAACATGTCCATGA -3'
*rs1048221*	5'- **ATGAAGTGCTAGGAATCCGC**CAAACATGTCCATGAGGGTCC -3'
*rs3750934*	5'- **TAATGGACATGACGCGACAG**AATTACAATCAGATGGGCCACA -3'
*rs8192466*	5'- **AAGTACGTCGAAGCAGCAGC**AGTTCCACCAGGTGAGAAGAGTGATGA -3'
*rs1047856*	5'- **AGCTCGATACTGACTGAGAT**GCCGTGGTACTCCGTGTGAT -3'
*rs2275857*	5'- **AGAGACGACCTAAGCCAGTC**TGGTAATGCTGTTTCTGCTTAAGTTG -3'
*rs2289656*	5'- **ATCTGGCGACGCTATGACGT**TCCTGGAGCCCACCTCCC -3'
*rs2289658*	5'- **CATAGATGGAAATCGGCGCA**CATCCTTCAGGGTCTGGGG -3'
*rs741072*	5'- **CCTCTGAGTAAATACGGAGA**GCATTCCCACACTGGCCC -3'
*rs2072446*	5'- **CTCCGGCATTAGAACATAAC**CTGGGGGCTGTGCTGTCC -3'
*rs2072445*	5'- **GTCCGCATTGCATGATGAGT**AGGAAGAACACGGCAGTGG -3'
*rs734194*	5'- **ATCATGTCACCAGAGTGCCG**CTCCACGTGTAAGCCCTTGA -3'

The sequence of primers used in the extension reactions is reported (in bold the Tag sequence). *Control *is the primer employed as positive hybridization control and it is labelled at the 5'-end (*) with either Cy3 or Cy5.

**Table 3:** SNP list

**GENE**	***SNP***	**ALLELES**	***LOCATION***
**NGFB**	*rs6330*	C/T	*Exon 1*
	*rs3738701*	C/A	*5'UTR*
	*rs11466112*	C/T	*Exon 1*
	*rs11466110*	G/A	*Exon 1*
	*rs11466111*	G/A	*Exon 1*
**NTRK1**	*rs6336*	C/T	*Exon 15*
	*rs6339*	G/T	*Exon 15*
	*rs1799770*	A/- (C)	*Intron 14*
	*rs3753213*	G/A	*Promoter Region*
	*rs1800878*	G/T	*Intron 2*
**BDNF**	*rs6265*	G/A	*Exon 1*
	*X60202*	C/T	*5'UTR*
	*rs1048218*	G/T	*Exon 1*
	*rs1048220*	G/T	*Exon 1*
	*rs1048221*	G/T	*Exon 1*
	*rs3750934*	G/A	*Intron 1*
	*rs8192466*	C/T	*Exon 1*
**NTRK2**	*rs1047856*	A/T	*Exon 8*
	*rs2275857*	G/C	*Exon 11*
	*rs2289656*	C/T	*Intron 15*
	*rs2289658*	G/A	*Intron 14*
**NGFR**	*rs741072*	C/T	*3'UTR*
	*rs2072446*	C/T	*Exon 4*
	*rs2072445*	G/T	*Intron 3*
	*rs734194*	T/G	*3'UTR*

A list of the genotyped SNPs (indicated with their NCBI database *rs*) is reported, according to the gene they belong to. For each SNP, the polymorphic alleles are indicated, as well as the gene region where they are located. All the SNPs in the exons give non-synonymous amino-acidic change. For the SNP X60202 its GeneBank code is reported.
