# Supplementary material for: TAMGeS: a Three-Array Method for Genotyping of SNPs by a dual-colour approach
Source: BMC Genomics. 2007 Jan 9;8:10. doi: 10.1186/1471-2164-8-10 (PMC1783851; doi:10.1186/1471-2164-8-10)
Supplement: Additional File 1 — TAMGeS theoretical basis. It is a PDF file and includes two sections, respectively entitled "Extracting information from the U-array" and "Applying bilinear regression", describing the mathematical foundations of the presented data handling method. [file 1471-2164-8-10-S1.pdf]

TAMGeS theoretical basis

(Silvia Giulia Galfré, Francesco Morandin, Roberto Marangoni)

Extracting information from the U-array

In the ideal case in which no noise perturbs the experimental procedures, named y_P the signal recorded in either the red or the green channel of a spot of the P-arrays, the corresponding signal recorded on the U-array should be:

$$y_U = y_{P_1} + y_{P_2} \tag{1}$$

Since different sources of noise act at different levels, we must take into account the possible systematic drift induced by the array quality and the performance of the experimental procedure. These errors can globally amplify or reduce the green and red signals of the whole array, in a spot-independent way, providing a multiplicative contribution. In each array, named x_i the real measured signal (red or green), and S_i the multiplicative error of that channel, we can write:

$$x_i = S_i y_i \tag{2}$$

Other sources of noise must be taken into account, which can be related to a specific channel (red or green) or to a specific spot of the array; their contribution, therefore, has a multiplicative (e_i) and an additive (ϵ_i) part. By rewriting equation 2 according to this:

$$x_i = (S_i + e_i)y_i + \epsilon_i \tag{3}$$

While S_i are systematic errors, e_i and ϵ_i are stocastic variables normally distributed. The mean values are supposed $\langle e_i \rangle = 0$ and $\langle \epsilon_i \rangle \approx b_{(r \text{ or } g)}$, respectively. We note that, for high values of x_i, it results $\epsilon_i \ll x_i$ and, as a consequence, additive errors have no significant influence on true signals.
One more necessary hypothesis, consistent with real data, is that the $\sigma_{e_i} \ll S_i$, this is to ensure that $(S_i + e_i) > 0$. These distributions have been applied and show the best fit with experimental data.
These considerations are applicable to all the arrays; therefore, by replacing the contents of Eq. 3 on Eq. 1 we obtain, for $i = \{P_1, P_2, U\}$:

$$\frac{x_U - \epsilon_U}{S_U + e_U} = \frac{x_{P_1} - \epsilon_{P_1}}{S_{P_1} + e_{P_1}} + \frac{x_{P_2} - \epsilon_{P_2}}{S_{P_2} + e_{P_2}} \tag{4}$$

After a few algebraic transformations, and assuming that it is possible to rule out any term with very low variance, we obtain an equation of the type:

$$x_U - \frac{S_U}{S_{P_1}} x_{P_1} - \frac{S_U}{S_{P_2}} x_{P_2} \approx e(x_{P_1}, x_{P_2}, x_U) + \epsilon' \tag{5}$$

where e gathers all the error terms which depend on the variables, and ϵ the others.
Named $a = \frac{S_U}{S_{P_1}}$ and $b = \frac{S_U}{S_{P_2}}$, we obtain:

$$x_U \approx a x_{P_1} + b x_{P_2} + e(x_{P_1}, x_{P_2}, x_U) + \epsilon' \tag{6}$$

This equation shows a form suitable for the application of bilinear regression theory. In the present case, we need to use weighted-least-squares procedure, and a prerequisite is to give an estimation of the values of the variance of both errors (e and ϵ').
We observe that the variance of ϵ' is negligible when computed on true signals (which are very strong): in fact, in this condition $x_i - \epsilon_i \approx x_i$, then the equation becomes:

$$x_U \approx a x_{P_1} + b x_{P_2} + e(x_{P_1}, x_{P_2}, x_U) \tag{7}$$

It is possible to estimate $\mathrm{Var}(e)$ using the independence of e_{P_1}, e_{P_2}, e_U. Named
$\sigma^2 = \mathrm{Var}(e_{P_1}) = \mathrm{Var}(e_{P_2}) = \mathrm{Var}(e_U)$, we have:

$$\mathrm{Var}(e) = \mathrm{Var}\left[\left(\frac{1}{S_{P_1} S_{P_2}}\right)\left(e_{P_1}(S_U x_{P_2} - S_{P_2} x_U) + e_{P_2}(S_U x_{P_1} - S_{P_1} x_U) + e_U(S_{P_2} x_{P_1} + S_{P_1} x_{P_2})\right)\right] \tag{8}$$

which turns into:

$$\mathrm{Var}(e) = \frac{\sigma^2}{S_{P_1}^2 S_{P_2}^2}\left\{(S_U x_{P_2} - S_{P_2} x_U)^2 (S_U x_{P_1} - S_{P_1} x_U)^2 + (S_{P_2} x_{P_1} + S_{P_1} x_{P_2})^2\right\}. \tag{9}$$

Expanding the squares, dividing by $S_{P_1} S_{P_2}$ the terms in curly brackets and replacing the values of a and b, we obtain:

$$\mathrm{Var}(e) = \frac{\sigma^2}{S_{P_1} S_{P_2}}\left\{x_{P_1}^2\left(ab + \frac{a}{b}\right) + x_{P_2}^2\left(ab + \frac{b}{a}\right) + x_U^2\left(\frac{a}{b} + \frac{b}{a}\right) + 2x_{P_1} x_{P_2} - 2b x_{P_1} x_U - 2a x_{P_2} x_U\right\} \tag{10}$$

Named $w(x_{P_1}, x_{P_2}, x_U)$ the weight, defined as the inverse of the term in curly brackets in the previous equation, we have:

$$\mathrm{Var}(e) = \frac{\sigma^2}{S_{P_1} S_{P_2} w(x_{P_1}, x_{P_2}, x_U)} \tag{11}$$

which allows us to apply bilinear regression technique.

Applying bilinear regression

By enumerating $1 \dots n$ the equations of the form (7) applicable to all the unambiguous signals, naming $x_{P_1,i}$ the intensity of the signal relative to the $i-$th spot on the P_1-array (the same for arrays P_2 and U), and indicating by w_i the weights relative to each equation, we can calculate the following coefficients:

$$\begin{array}{lll} c_{11} = \sum_{i=1}^{n} w_i x_{P_1,i}^2 & c_{12} = \sum_{i=1}^{n} w_i x_{P_1,i} x_{P_2,i} & c_{22} = \sum_{i=1}^{n} w_i x_{P_2,i}^2 \\ d_1 = \sum_{i=1}^{n} w_i x_{P_1,i} x_{U,i} & d_2 = \sum_{i=1}^{n} w_i x_{P_2,i} x_{U,i} & \end{array} \tag{12}$$

We can then write the *normal equations* of the model and find the estimators for a and b, (named $\hat{a}$ and $\hat{b}$, respectively) by computing:

$$\hat{a} = \frac{c_{22} d_1 - c_{12} d_2}{c_{11} c_{22} - c_{12}^2} \qquad \hat{b} = \frac{c_{11} d_2 - c_{12} d_1}{c_{11} c_{22} - c_{12}^2} \tag{13}$$

An important difference between the standard method of bilinear regression and the present instance of application emerges from the observation that the weights depend on the parameters a and b. This point can be managed by following an iterative approach:

a) start by assuming $a_0 = b_0 = 1$;

b) compute the corresponding weights;

c) execute the bilinear regression procedure;

d) extract the estimated values $\hat{a}$ and $\hat{b}$;

e) repeat from the point b);

This process stops when the difference between the values obtained for $\hat{a}$ and $\hat{b}$ at the n-th cycle and those obtained at the $(n-1)$-th cycle is smaller than a previously defined precision threshold. We have empirically verified that, in our real experiments, the convergence is reached with only 2-3 iteration cycles.
