# Supplementary material for: TAMGeS: a Three-Array Method for Genotyping of SNPs by a dual-colour approach
Source: BMC Genomics. 2007 Jan 9;8:10. doi: 10.1186/1471-2164-8-10 (PMC1783851; doi:10.1186/1471-2164-8-10)
Supplement: Additional File 2 — It is a PDF file, including three graphs respectively on amount of unsolvable signals, allelic and genotypic call rates: Graph 1 – Unsolvable signals. Graph 2 – Non-assigned alleles. Graph 3 – Non-assigned genotypes. [file 1471-2164-8-10-S2.pdf]

Graphs

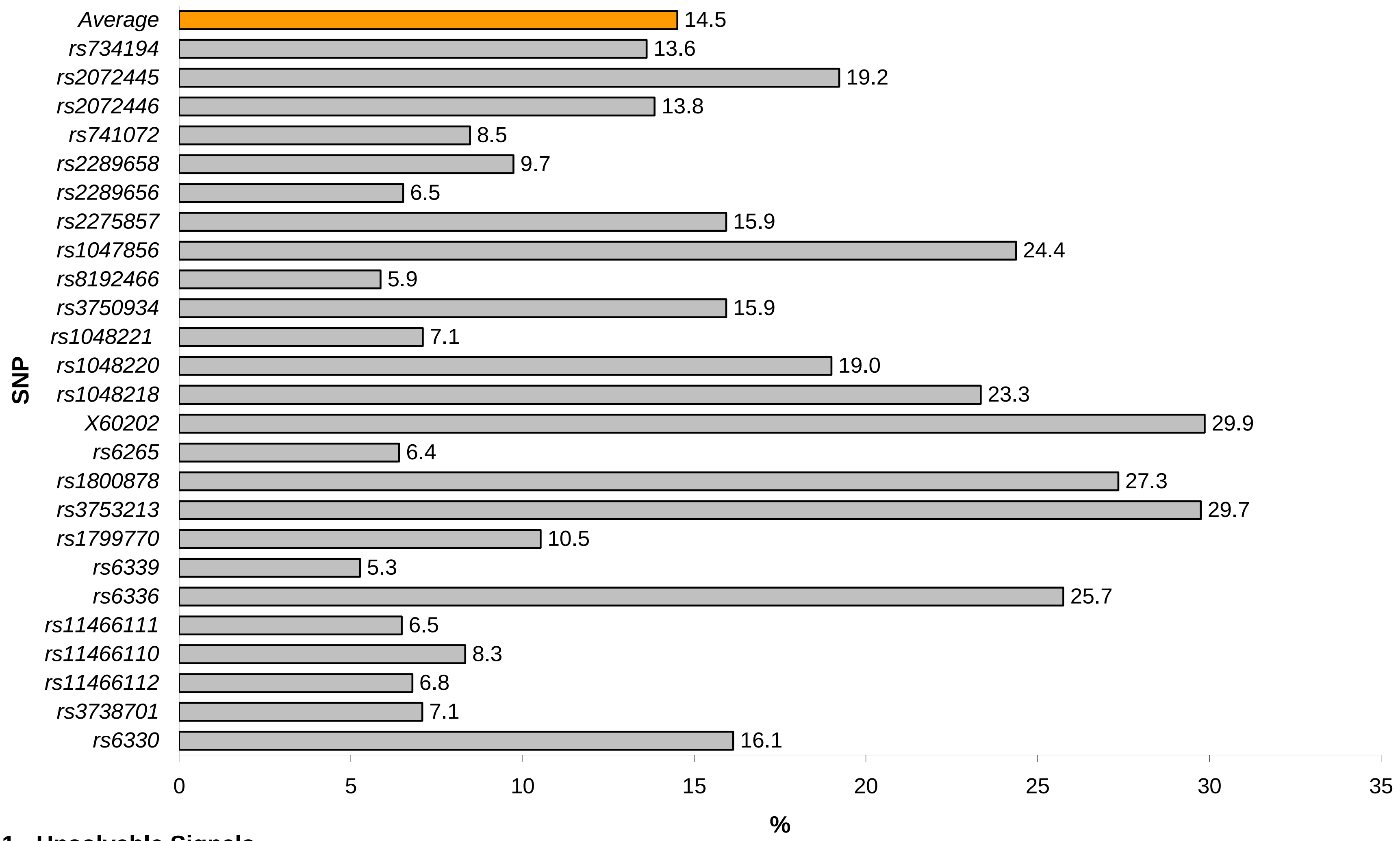

Graph 1 - Unsolvable Signals

For each SNP (indicated by its *rs*), the percentage of unsolvable signals on the total amount of expected signals (according to the number of analysed samples) is plotted. The average percentage of unsolvable signals is reported in orange.

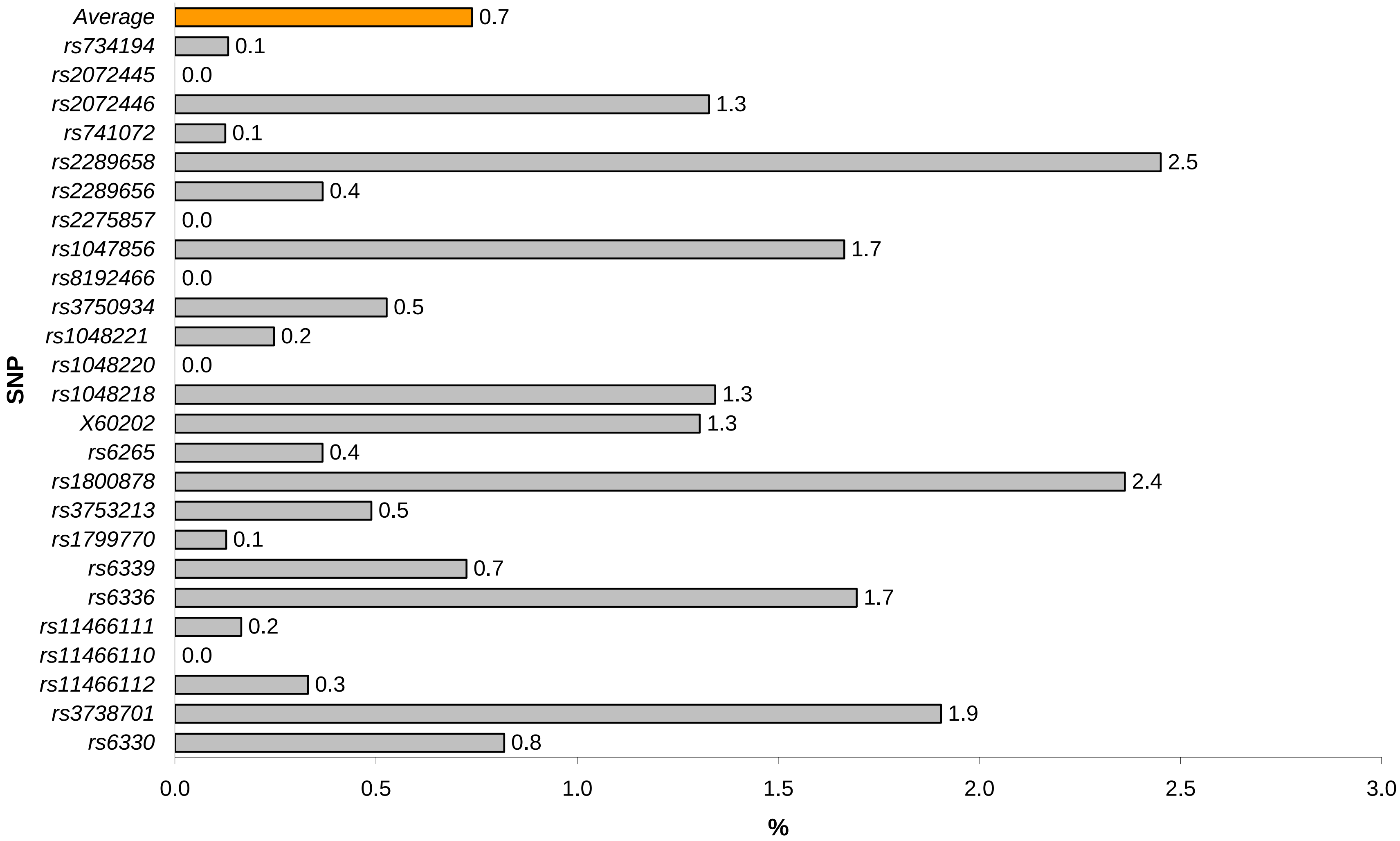

Graph 2 - Non-assigned alleles

For each SNP (indicated by its *rs*), the percentage of alleles we could not call (see Methods, Data Analysis - *Third Module: genotype and allele assignment*) respect to the total amount of analyzable signals is plotted. The average percentage of non-assigned alleles is reported in orange.

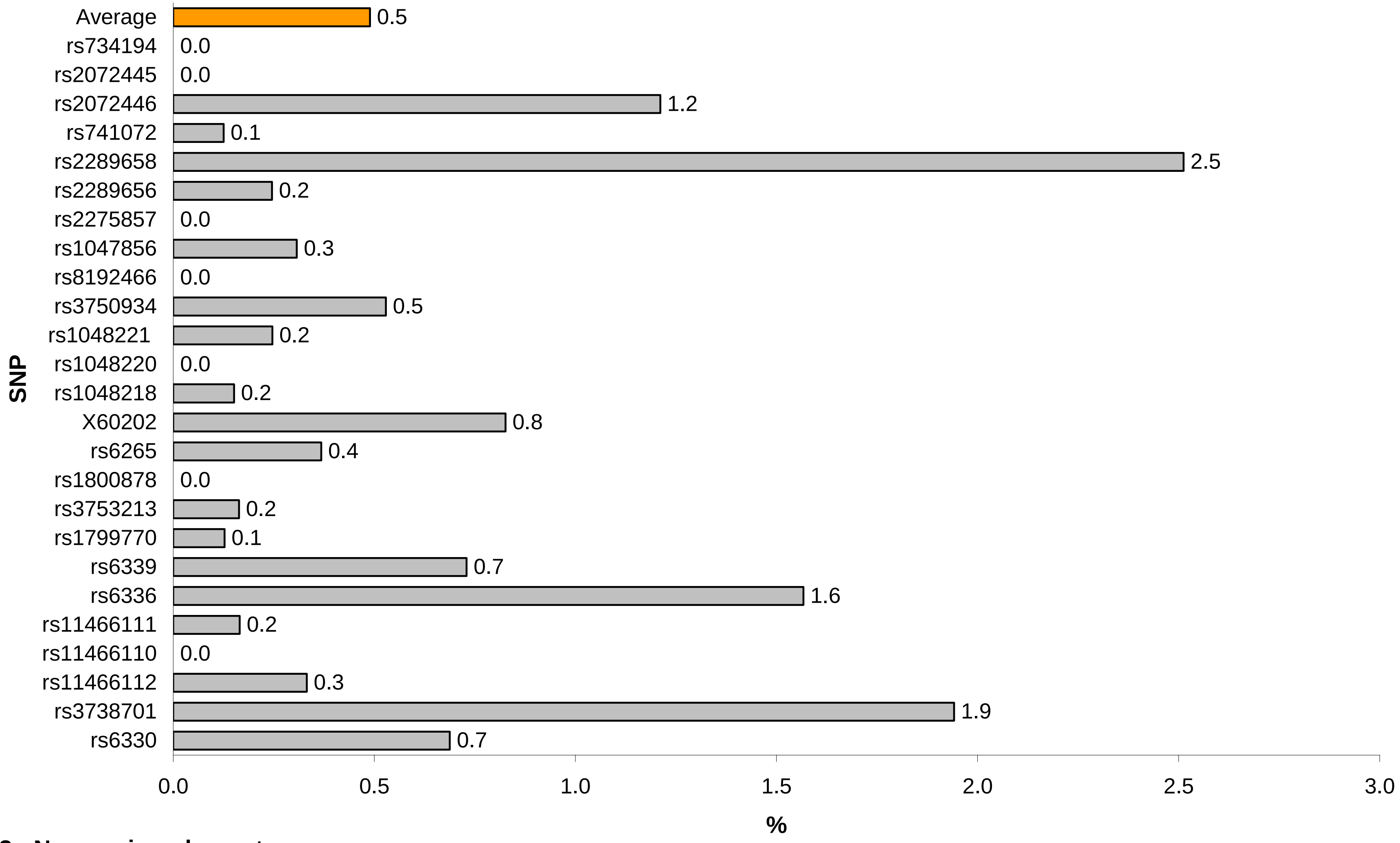

Graph 3 - Non-assigned genotypes

For each SNP (indicated by its *rs*), the percentage of genotypes we could not call (see Methods, Data Analysis - *Third Module: genotype and allele assignment*) respect to the total amount of expected genotypes (calculated from the amount of called alleles) is plotted. The average percentage of non-assigned genotypes is reported in orange.
